# Anti-Inflammatory and Neuroprotective Role of Natural Product Securinine in Activated Glial Cells: Implications for Parkinson's Disease

**DOI:** 10.1155/2017/8302636

**Published:** 2017-04-04

**Authors:** Dmitri Leonoudakis, Anand Rane, Suzanne Angeli, Gordon J. Lithgow, Julie K. Andersen, Shankar J. Chinta

**Affiliations:** ^1^Buck Institute for Research on Aging, 8001 Redwood Blvd, Novato, CA 94945, USA; ^2^Touro University California College of Pharmacy, 1310 Club Drive, Vallejo, CA 94592, USA

## Abstract

Glial activation and subsequent release of neurotoxic proinflammatory factors are believed to play an important role in the pathogenesis of several neurological disorders including Parkinson's disease (PD). Inhibition of glial activation and inflammatory processes may represent a therapeutic target to alleviate neurodegeneration. Securinine, a major natural alkaloid product from the root of the plant* Securinega suffruticosa*, has been reported to have potent biological activity and is used in the treatment of neurological conditions such as amyotrophic lateral sclerosis, poliomyelitis, and multiple sclerosis. In this study, we explored the underlying mechanisms of neuroprotection elicited by securinine, particularly its anti-inflammatory effects in glial cells. Our results demonstrate that securinine significantly and dose-dependently suppressed the nitric oxide production in microglia and astrocytic cultures. In addition, securinine inhibited the activation of the inflammatory mediator NF-*κ*B, as well as mitogen-activated protein kinases in lipopolysaccharide- (LPS-) stimulated BV2 cells. Additionally, securinine also inhibited interferon-*γ*- (IFN-*γ*-) induced nitric oxide levels and iNOS mRNA expression. Furthermore, conditioned media (CM) from securinine pretreated BV2 cells significantly reduced mesencephalic dopaminergic neurotoxicity compared with CM from LPS stimulated microglia. These findings suggest that securinine may be a potential candidate for the treatment of neurodegenerative diseases related to neuroinflammation.

## 1. Introduction

Inflammation of the central nervous system (CNS) is a key contributing factor in several neurodegenerative diseases. Glial cells, including microglia and astrocytes, are known to be the major mediators of neuroinflammation. Activated microglial cells trigger an inflammatory response that is then maintained and often times amplified by astrocytes. This, in turn, exposes neurons to inflammatory mediators that can cause neuronal cell death [1]. Neurodegenerative CNS diseases including Alzheimer's disease (AD), Parkinson's disease (PD), Huntington's disease (HD), amyotrophic lateral sclerosis (ALS), and age-related macular degeneration (ARMD) have all been associated with chronic neuroinflammation and activation of both microglia and astrocytes [2]. This chronic inflammation can lead to the accumulation of neurotoxic molecules including proinflammatory cytokines, proteinases, and reactive oxygen species (ROS), which participate in the neurodegenerative process and can ultimately lead to neuronal cell death [3–5]. It is hypothesized that inhibiting microglial-mediated induction of the neuroinflammatory process may prove to be neuroprotective and therefore inhibitory compounds targeting this process are likely to have therapeutic value.

Medicinal plants and their active components are currently the subject of extensive biomedical research. Many of these plant-derived medications have been validated by traditional usage over the centuries, but the specific bioactive components have not been identified or are not fully characterized. Traditional herbal medicines with neurotrophic and neuroprotective properties have been demonstrated to prevent neurodegeneration and neuroinflammatory diseases [6]. This suggests that these traditional medicinal herbs possess neuroprotective benefits through distinct and multiple mechanisms, including anti-inflammation [7–10]. Natural compounds specifically targeted to blocking microglial activation and therefore blocking the initiation of CNS inflammation may prove to be an effective therapy to prevent neurodegenerative and neuroinflammatory diseases.

Securinine, a major natural alkaloid product from the root of the plant* Securinega suffruticosa,* has been reported to have potent biological activity and has been used clinically in several countries [11]. Securinine has been found to be an antagonist of the *γ*-amino butyric acid (GABA) A receptor and as such is currently used in the treatment of neurological conditions such as ALS, poliomyelitis, and multiple sclerosis [12–14]. Additionally, securinine has been shown to be a macrophage agonist, enhancing bacterial clearance via a mechanism distinct from toll-like receptors (TLRs) [15]. Securinine appears to stimulate p38 MAPK activity through antagonism of the GABA A receptor in monocytes [16]. Securinine has also been found to stimulate apoptosis in p53-null colon cancer cells [1, 17]. However, the potential anti-inflammatory role of securinine on glial activation and the mechanisms involved have not been thoroughly explored.

In the present study, to determine whether securinine can suppress glial inflammatory activation and act as a mediator of neuroinflammation, we tested the effects of securinine on lipopolysaccharide- (LPS-) induced activation of microglia and astrocytes. The results demonstrated that securinine inhibits the activation of the inflammatory mediator NF-*κ*B, as well as its upstream MAPK activators including ERK. Further, securinine also silences iNOS expression and NO production, both of which are activated by NF-*κ*B. Importantly, we demonstrate that inhibition of LPS-induced microglial activation with securinine is neuroprotective in the context of primary mesencephalic dopaminergic neurons representing an in vitro model of PD.

## 2. Experimental Procedures

### 2.1. Cell Culture

The cell culture reagents used in this study were purchased from Mediatech, Inc. (Manassas, VA, USA). BV2 microglial cells were cultured in Dulbecco's modified Eagle medium (DMEM) containing 10% fetal bovine serum (FBS) and 1% penicillin-streptomycin mixture (10,000 u/mL) (Sigma Aldrich, St Louis, MO). RAW 264.7 macrophage cell lines (ATCC, TBI-71) were grown and maintained in DMEM supplemented with 10% heat inactivated FBS and 1% Penicillin-streptomycin mixture (10,000 u/mL) at 37°C, 5% CO_2_.

Primary mouse astrocytes and microglia were isolated as described previously [18]. We used postnatal day 2 pups to generate primary glial cultures. Briefly, cortical tissues from C57/BL6 mice were dissected out and the tissue was digested with papain for 30 minutes at 37°C and plated in poly-L-lysine coated T75 flasks in DMEM supplemented with 10% FBS. For astrocytes culture isolation, after 1 week of culture, the flasks were shaken to remove microglia followed by trypsinization and plating onto poly-L-lysine coated plates or chambered slides at 1 × 10^5^ cells/well. The cells were only used for a maximum of three passages; more than 95% of cells stained positively for the astrocyte marker GFAP in these cultures.

Primary microglial cultures were prepared from the cortex of C57/BL6 mice as described above. Briefly, mixed glial cells were cultured for 3-4 weeks in DMEM supplemented with 10% FBS. The astrocyte monolayer was removed by incubation for 30 min with 0.25% trypsin/2.12 mM EDTA (Mediatech, Inc., Manassas, VA) diluted 1 : 4 with DMEM. The remaining isolated microglial cells were plated into 96-well plates at a density of 5 × 10^4^ cells/well and 6-well plates at 5 × 10^5^ cells/well. The purity of microglia cultures was assessed using microglial marker CD11b or Iba1; 95% of cells stained positively for these microglial markers; 1–3% stained positively for GFAP marker. The microglial cells were cultured for 2 days before drug treatment.

Primary mesencephalic cultures were isolated as previously described from our laboratory [19]. Briefly, ventral mesencephalon was dissected from embryonic day 14 of C57/BL6 mice. The tissue was dissociated mechanically and then digested enzymatically with papain solution as per manufactures instructions (Worthington Biochemical, Lakewood, NJ). After 4 d in vitro, the neuronal cultures were taken for condition media exposure from activated microglial cells.

### 2.2. Cell Viability Assay

Cell viability was determined by calorimetric MTT (3-(4,5-dimethylthiazol-2-yl)-2,5-diphenyltetrazolium bromide) assay as described previously from our laboratory [20]. BV2 microglial cells were plated into 96-well culture plates at a density of 1 × 10^4^ cells/mL in 100 *μ*L culture medium per well. The cells were allowed to settle for 24 h before the addition of different concentrations of securinine for 24 h (Sigma Aldrich, USA). After 24 h of treatment, MTT solution (5 mg/mL) was added to each well and plate was incubated at 37°C for 2–4 h. The medium was aspirated and the resulting formazan crystals were dissolved in 200 *μ*L of dimethyl sulfoxide before measuring the absorbance at 570 nm using a spectrophotometer.

### 2.3. Measurement of Nitrite (NO2^−^) Levels

The level of NO2^−^, a stable downstream product of NO, secreted by activated glial cells in culture media was measured using the Griess Reagent assay method as previously described [21]. The microglia, astrocytes, and macrophages cultures were exposed to inflammation inducing agents in presence or absence of different concentration of securinine for 24 h. Following the treatments, NO levels in the culture media were measured by mixing with equal volume of Griess Reagent and OD was measured at 540 nm on a microplate reader (Molecular Devices, CA). The data are representative of results obtained from four independent experiments performed in triplicate (mean ± SD).

### 2.4. NF*κ*B-Luciferase Activity Assay

BV2 cells stably expressing an NF*κ*B-luciferase construct were plated into 96-well cell culture plates at a density of 10,000 cells/well. The NF*κ*B binding reporter plasmid contains three copies of the *κ*B-binding sequence fused to the firefly luciferase gene which was purchased from Clontech (Mountain View, CA, USA). Cells were preexposed to securinine followed by cotreatment with LPS for 24 hr treatment. Luciferase activity was measured using the Dual-Luciferase® Reporter Assay System kit as per the manufacturer's instructions (Promega).

### 2.5. Western Blotting Analysis

Following various treatments, BV2 cells and astrocytes were collected from Petri dishes by trypsinization and lysed in cold lysis buffer containing protease inhibitors as previously described [21]. The whole cell lysates were sonicated and the protein concentration was measured using a Bradford protein assay kit (Bio-Rad, Hercules, CA). Twenty-five to 50 *μ*g of total protein was subjected to 10% SDS-PAGE and protein was transferred to a polyvinylidene difluoride membrane. Immunodetection was carried out by standard procedures using the following dilutions of antibodies: iNOS (rabbit polyclonal antibody, 1 : 1000), phospho-NFkB (p65) (rabbit polyclonal antibody, 1 : 1000), PARP (rabbit polyclonal antibody, 1 : 1000), the phospho- or total forms of ERK1/2, p38 MAPK, JNK, and pSTAT1 (rabbit polyclonal antibodies, 1 : 1000, Cell Signaling Technology, USA), and beta-actin (mouse monoclonal antibody, 1 : 10,000, Sigma Aldrich, USA). Secondary antibodies were horseradish peroxidase conjugated to either goat anti-rabbit IgG or anti-mouse IgG (Cell Signaling Technology, USA). The membranes were developed using an enhanced chemiluminescence (ECL) detection system (Pierce, USA). Band intensities were determined using the Image-Pro Plus 6.0 software (Bio-Rad).

### 2.6. Quantitative Polymerase Chain Reaction (qPCR) Analysis

BV2 cells and primary astrocytes cultured were challenged with LPS (BV2, 500 ng/mL; primary astrocytes, 1 *μ*g/mL + IFN-*γ* 40 U/mL) in the presence or absence of securinine (10 *μ*M) for 6 h. Total RNA was isolated from the cells via TRIzol method and cDNA was synthesized using Promega GoScript™ Reverse Transcription System (Madison, WI, USA). Quantitative PCR was performed using SYBR Green PCR Master Mix reagent and gene-specific primers. Data were analyzed by using the comparative threshold cycle (Ct) method. Altered mRNA expression levels following treatment were calculated following normalization to GAPDH. The ratios obtained after normalization are expressed as fold change versus corresponding controls [20].

### 2.7. Neurotoxicity of BV2 Conditioned Media in Primary Mesencephalic Cultures

Primary mesencephalic cultures were isolated as described above. After 4 days in vitro, dopaminergic neurons were exposed to conditioned media (CM) from BV2 microglial cells treated with LPS ± securinine. The CM was prepared as follows: BV2 cells cultured in 6-well plates were stimulated with LPS (500 ng/mL) in the presence and absence of securinine (10 *μ*M) for 24 h. CM was collected from all the groups and centrifuged at 2,000 g for 10 min to remove any cell debris. The CM from control, securinine, and LPS versus LPS + securinine-treated cells was diluted (1 : 4) in neuronal culture media before adding to mesencephalic cultures. After 72 h exposure, the cells were fixed in 4% paraformaldehyde and immunostained with anti-TH antibody and Alexa Fluor 488 secondary antibody. Total numbers of TH-positive neurons were counted in 3–5 separate wells for each condition. Experiments were repeated with cultures isolated from three independent dissections.

### 2.8. Statistical Analysis

Unless otherwise stated, all experiments were performed in triplicate and repeated at least three times. The data are presented as mean ± SEM and statistical comparisons between groups were performed by one-way ANOVA followed by Student's *t*-test. Multiple comparisons of data from in vitro experiments were evaluated by two-way ANOVA followed by Bonferroni post hoc testing. Statistical significance was set at *P* < 0.05 for all analyses.

## 3. Results

### 3.1. Securinine Does Not Elicit Cellular Toxicity in Microglial BV2 Cells at Concentrations up to 15 *μ*M

**Figure 1:**
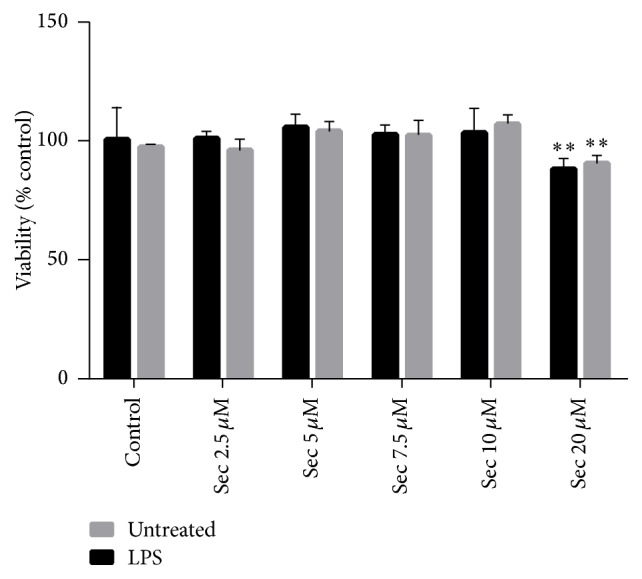
Effects of securinine on the cell viability of the BV2 microglia cell line. BV2 cells were treated with 2.5–20 *μ*M of securinine without LPS treatment or in the presence of 500 ng/mL LPS treatment for 24 h. Cell viability was measured via the MTT assay and data was expressed as mean ± SEM for three independent experiments. ^*∗∗*^*P* < 0.05, significantly different from the value in control cells.

To determine whether securinine displays cytotoxic effects in glial cell types, we treated BV2 cells, an immortalized murine microglial cell line, with increasing concentrations of the compound for 24 h, followed by assessment of cell viability using the MTT assay. No significant cytotoxicity was observed up to 15 *μ*M, whereas 15% toxicity was observed at 20 *μ*M (Figure 1). This toxicity may be due to the induction of apoptosis as previously reported at 30 *μ*M in cancer cell lines [17]. Based on this data, we chose to use 10 *μ*M securinine for all subsequent assays. Additionally, the concentration of LPS (500 ng/mL) used to induce NO production also did not affect cell viability (data not shown).

### 3.2. Securinine Inhibits LPS-Induced Inflammatory NO Production in Both BV2 Cells and Primary Mouse Microglia

**Figure 2:**
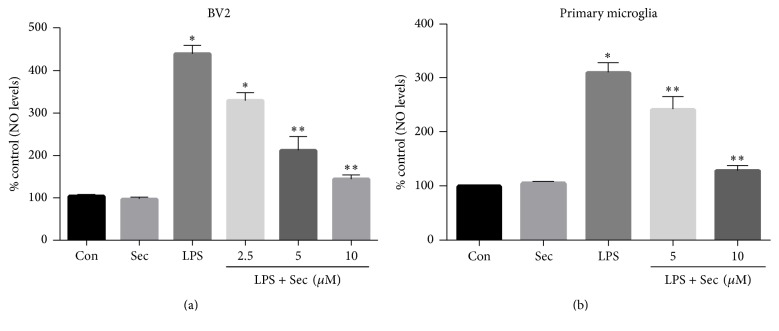
Securinine inhibits LPS-induced NO production in the BV2 cell line and in mouse primary microglia challenged with LPS exposure. BV2 microglial cells (a) and mouse primary microglia (b) were pretreated with securinine (2, 5, and 10 *μ*M) for 1 h and then stimulated with LPS (500 ng/mL) for 24 h. NO levels in the medium were determined using the Griess Reagent system. The data are expressed as mean ± SD, *n* = 4. ^*∗*^*P* < 0.01, significantly different from control samples. ^*∗∗*^*P* < 0.05 significantly different from the LPS-treated group alone.

To examine the potential anti-inflammatory activity of securinine in the context of microglial activation, we initially studied the effects of securinine on the production of inflammatory mediators in BV2 and mouse primary microglia, both challenged with LPS. As part of the inflammatory response, activated glial cells rapidly induce the expression of inducible nitric oxide synthase (iNOS), a key enzyme required for generating nitric oxide (NO), a reactive nitrogen species (RNS) causing protein and mitochondrial damage leading to apoptosis [22]. In BV2 cells, LPS markedly induced NO production as detected by the accumulation of nitrite in the culture medium after 24 h. In the presence of securinine however, there was a dose-dependent inhibition of NO production to almost control levels at the highest drug concentration. Securinine alone had no effect on NO production (Figure 2(a)). Likewise, we observed significant inhibition of LPS-induced NO production by securinine within primary microglia cell cultures (Figure 2(b)).

### 3.3. Securinine Inhibits LPS-Induced Expression of the NO-Synthesizing Enzyme, iNOS, and Proinflammatory Cytokines

**Figure 3:**
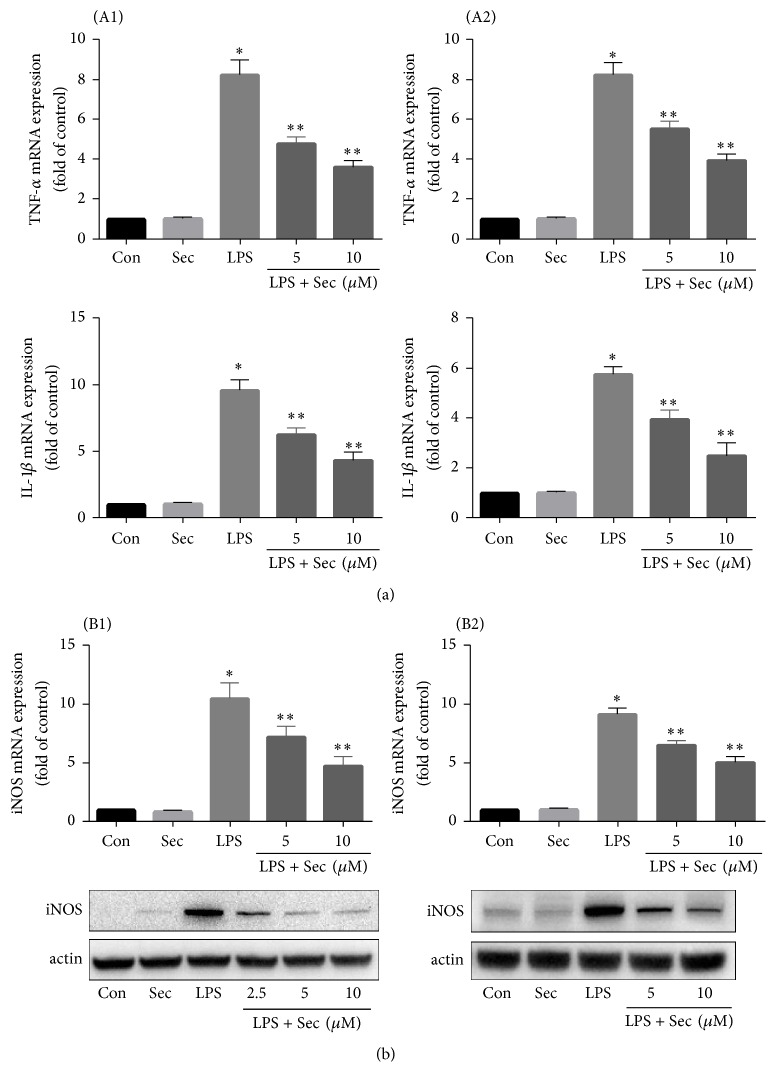
Securinine represses LPS-induced proinflammatory cytokines and iNOS expression in BV2 and mouse primary microglia. (a) Securinine inhibits proinflammatory cytokine expression following LPS exposure. BV2 and primary microglial cells were preincubated with securinine for 1 h before stimulation with LPS (500 ng/mL). After 6 h of stimulation, total RNA was isolated and levels of mRNA expression of proinflammatory cytokines TNF-*α* and IL-1*β* analyzed via quantitative real-time PCR ((A1) BV2 cells and (A2) mouse primary microglia). (b) Securinine represses LPS-induced iNOS mRNA and protein expression in BV2 (B1) and primary microglia cells. After 24 h of stimulation with LPS (500 ng/mL), the level of iNOS protein was monitored by Western blot analysis ((B1) BV2 cells and (B2) primary microglia cells). Data are mean ± SD of three different experiments. ^*∗*^*P* < 0.001 versus control; ^*∗∗*^*P* < 0.05 versus LPS-treated group alone.

To examine whether the reduction of NO production by securinine was due to reduced mRNA and protein expression of iNOS and also effect of securinine on the expression of various proinflammatory cytokines, real-time PCR and Western blot analyses were conducted in LPS-stimulated BV2 and primary microglial cells. Both primary and BV2 cells were preincubated 1 h with securinine (10 *μ*M) and then stimulated for 6 h with LPS (500 ng/mL). As shown in Figures 3(A1) and 3(A2), securinine significantly reduced the expression of proinflammatory molecules such as TNF-*α* and IL-1*β* in both BV2 and primary microglial cells, while the compound alone did not induce any significant changes in gene expression. Furthermore, as shown by qPCR and Western blotting (Figures 3(B1) and 3(B2)), securinine dose-dependently inhibited LPS-induced iNOS expression at the mRNA and protein levels in both primary BV2 and microglial cells.

### 3.4. Securinine Attenuates LPS-Induced NF*κ*B Activation

**Figure 4:**
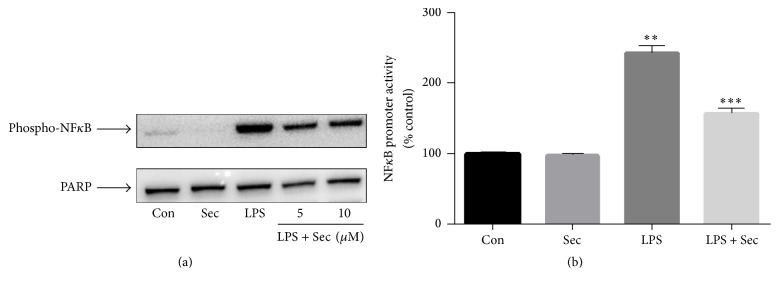
Securinine attenuates LPS-induced activation of NF-*κ*B in BV2 microglia. (a) BV2 microglial cells were treated with securinine for 1 h followed by stimulation with 500 ng/mL LPS. After 30 min of LPS stimulation, isolated nuclear lysates were subjected to Western blotting to assess translocation of phosphor-65 protein to the nucleus. PARP was used as a nuclear loading control. (b) BV2 cells were transiently transfected with NF-*κ*B-Luc plasmid for 24 h and then treated with 500 ng/mL LPS for 4 h ± securinine. Whole cell lysates were assayed via the luciferase activity (mean ± SE, *n* = 4); ^*∗*^*P* < 0.05 versus control. ^*∗∗*^*P* < 0.05 versus LPS. ^*∗∗∗*^*P* < 0.05 versus LPS.

LPS and other inflammatory stimuli are known to induce iNOS expression in glia cells via the activation of the transcription factor NF*κ*B. Therefore, we examined whether the anti-inflammatory effects of securinine were due to blockade of NF-*κ*B activation within BV2 cells. NF-*κ*B is normally activated by phosphorylation of I*κ*B proteins, targeting them for rapid degradation through the ubiquitin-proteasome pathway and releasing NF-*κ*B to enter the nucleus where it regulates gene expression. Western blot analysis demonstrated LPS-induced phosphorylation of p65 subunit which was significantly inhibited by pretreatment with securinine (Figure 4(a)). Furthermore, LPS-induced NF*κ*B-dependent transcriptional activity was also significantly reduced by treatment with securinine (10 *μ*M). These data suggest that securinine can prevent NF*κ*B-mediated induction of inflammatory pathways.

### 3.5. Securinine Reduces LPS-Induced MAPK Phosphorylation

**Figure 5:**
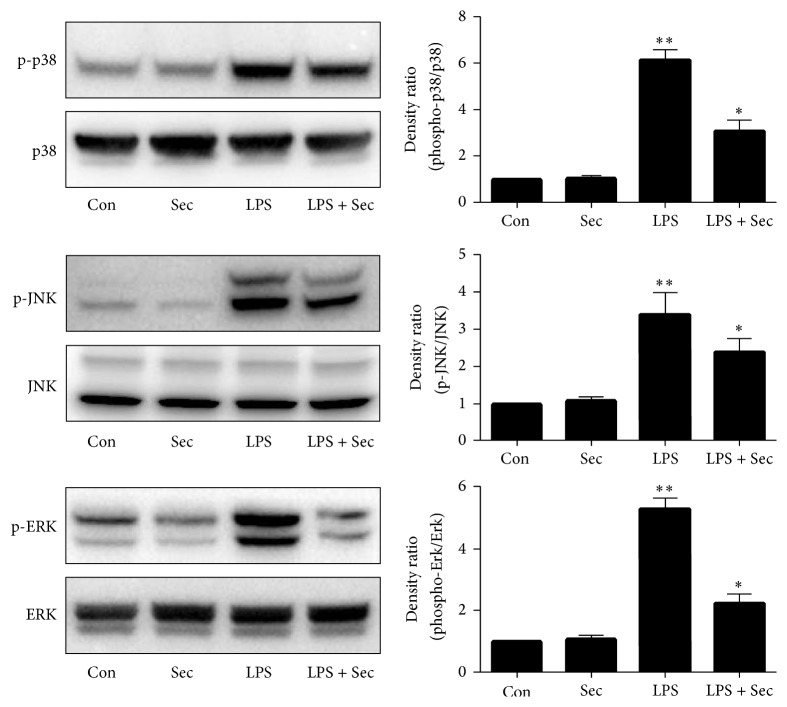
Securinine reduces LPS-induced MAPK phosphorylation in BV2 microglial cells. BV2 cells were pretreated with securinine (10 *μ*M) for 1 h and then exposed to LPS (500 ng/mL) for 30 minutes. Whole cell lysates (50 *μ*g of protein) were subjected to Western blot analysis using antibodies specific for phosphorylated forms of p38MAPK, ERK1/2, and JNK (mean ± SE, *n* = 4). Equal loadings of cell lysates were confirmed by reprobing the blots with total p38MAPK, ERK1/2, and JNK antibodies. The quantification data are shown in the right panel. ^*∗∗*^*P* < 0.01 versus control; ^*∗*^*P* < 0.05 versus LPS.

Inhibitors targeting MAPK signaling pathways have been known to exhibit anti-inflammatory activity [23]. To determine whether anti-inflammatory activity of securinine is due to modulation of MAPKs activity, BV2 cells were pretreated with securinine (10 *μ*M) for 1 h and then stimulated with LPS for an additional 1 h incubation period. Based on preliminary time course studies (data not shown), 1 h treatment of LPS was determined to be optimal for MAPK phosphorylation. Western blot analysis was carried out using phosphoantibodies or total antibodies against the three MAPKs, p38 MAPK, JNK, and ERK1/2. Western blot analysis showed that the securinine significantly repressed the phosphorylation of p38 MAPK, JNK, and ERK1/2, respectively, but did not affect the expression levels of ERK1/2, JNK, and p38 MAPK in LPS-stimulated BV2 microglia (Figure 5).

### 3.6. Securinine Reduces IFN-*γ*-Induced NO Production, iNOS mRNA Expression, and STAT1*α* Activation

**Figure 6:**
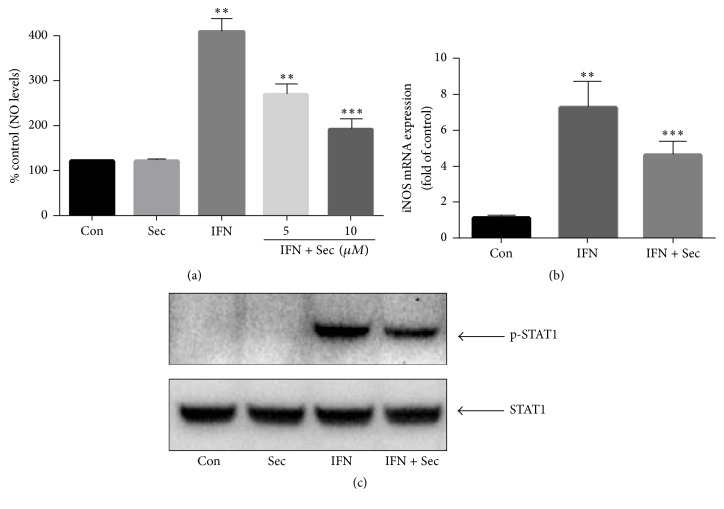
Securinine pretreatment reduces IFN-*γ*-induced NO production, iNOS mRNA expression, and phosphorylation of STAT1*α* in BV2 microglia. BV2 microglial cells were pretreated with securinine for 1 h and then challenged with IFN-*γ* (50 U/mL) for 24 h (a), 6 h (b), or 30 min (c). The expression levels of nitric oxide, iNOS mRNA, and tyrosine phosphorylation of STAT1*α* were determined by Griess Reagent assay (a), real-time PCR analysis (b), or Western blotting (c), respectively. The data are expressed as mean ± SD, *n* = 4. ^*∗∗*^*P* < 0.01 significantly different from control samples. ^*∗∗∗*^*P* < 0.05, significantly different from the LPS-treated group.

Multiple transcription factors participate in the regulation of iNOS promotor activity. In addition to LPS, IFN-*γ* is a well-established stimulus that promotes the expression of inflammatory molecules (e.g., iNOS) through STAT1*α*, but independent of NF-*κ*B [23]. Thus, the effects of securinine on IFN-*γ* activation were also investigated. As shown in Figure 6(a), securinine inhibited IFN-*γ*-induced NO production in a concentration-dependent manner with maximum inhibition achieved at 10 *μ*M. Securinine also significantly reduced IFN-*γ*-induced iNOS mRNA expression levels (Figure 6(b)) and phosphorylation of STAT1*α* (Figure 6(c)).

### 3.7. Securinine Protects Neurons through Inhibition of Microglial Activation

**Figure 7:**
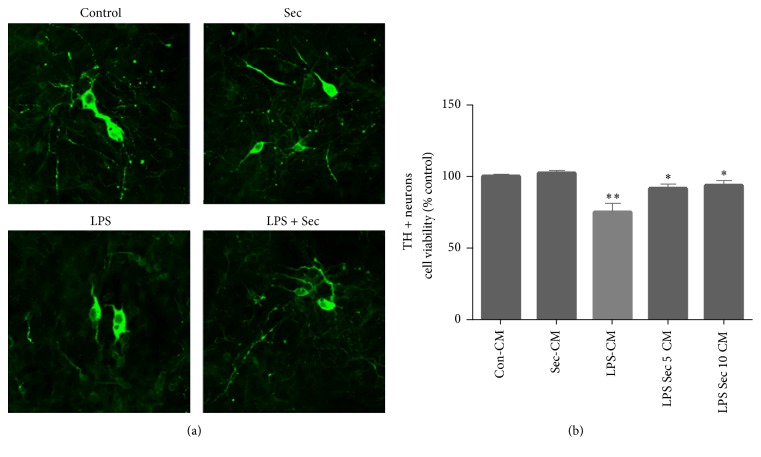
Securinine protects primary mesencephalic dopaminergic neurons through inhibition of BV2 cells activation. BV2 cells were stimulated with LPS (500 ng/mL) ± securinine (10 *μ*M) for 24 h. CM from control (BV2-CM), securinine-treated (Sec-CM), LPS-treated (LPS-CM), and LPS/securinine-treated (5 and 10 *μ*M; LPS + Sec 5 *μ*M-CM, LPS + Sec 10 *μ*M-CM) BV2 cells was added to primary mesencephalic cultures. After 72 h, cultures were immunostained for TH^+^ neurons (a) and the number of TH^+^ neurons was counted (b). Data are expressed as mean ± SD, *n* = 4; ^*∗∗*^*P* < 0.01, compared with control-CM group, ^*∗*^*P* < 0.05, compared with LPS-CM group.

Previous studies from our own laboratory and others have demonstrated that activated microglia induce neural cell death and amplify the progression of neuronal degeneration [20, 24]. Since our data suggested that securinine can suppress microglial activation, we investigated whether this translated to noncell autonomous neuroprotective effects. Conditioned media (CM) from LPS-treated BV2 cells in the presence or absence of securinine pretreatment were added to primary mesencephalic cultures and cell viability monitored in the latter via tyrosine hydroxylase (TH) immunocytochemistry and TH-positive neuronal counting (Figures 7(a) and 7(b)). Results from these studies demonstrated that CM from LPS-stimulated microglia (LPS-CM) showed significant toxicity to TH-positive neurons, presumably as a consequence of toxic factors secreted by the activated microglia. In addition to cell loss, neuritic processes were also shortened in surviving TH-positive neurons (Figure 7(b)). Securinine alone, under normal conditions, did not influence TH-positive viability. However, pretreatment of microglial cells with securinine provided significant neuroprotection against toxicity associated with CM isolated from the activated microglia (LPS/Scu-CM versus LPS-CM), suggesting that securinine may exert its neuroprotective effects, at least partly, via reducing the production and secretion of inflammatory mediators from the microglia (Figure 7(b)).

### 3.8. Effects of the Securinine on the Inflammatory Activation of RAW 264.7 Macrophage Cells and Primary Astrocyte Cultures

**Figure 8:**
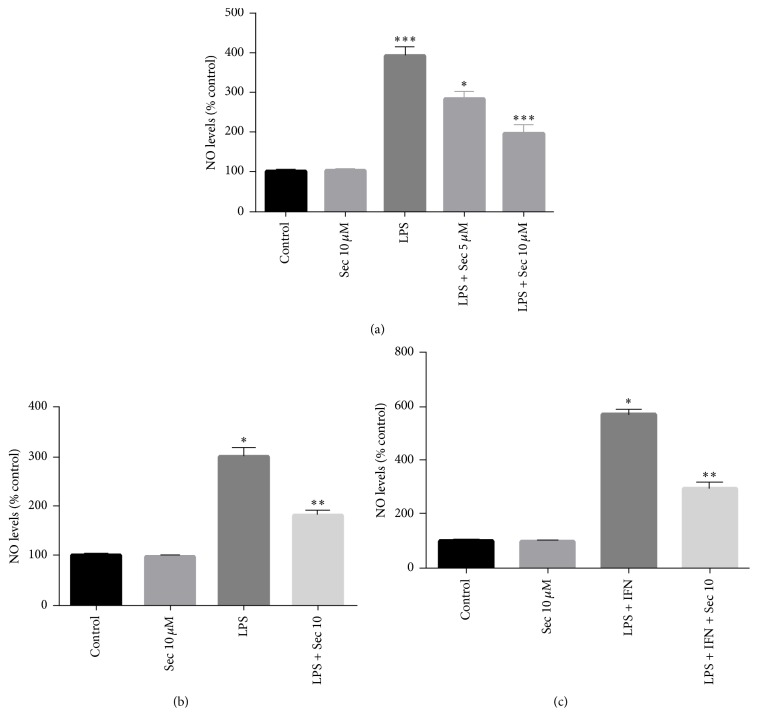
Securinine pretreatment inhibits LPS-induced NO production in RAW 264.7 macrophage cells and in primary astrocyte cultures. RAW 264.7 macrophage cells or primary astrocyte cultures (10 × 10^4^ cells/well in 48-well plates) were incubated with 500 ng/mL of LPS or LPS/IFN-*γ* (50 unit/mL) in the presence or absence of securinine (10 *μ*M) for 24 h. The amount of nitrite secreted into the supernatant was measured using the Griess Reagent assay ((a) RAW 264.7 cells, ^*∗∗∗*^*P* < 0.05 versus control; (b and c) primary astrocyte cultures). The data were expressed as the mean ± SD (*n* = 3) and are representative of three independent experiments. ^*∗*^*P* < 0.05 and ^*∗∗*^*P* < 0.01, significantly different from cells treated with LPS only.

Finally, in order to explore whether securinine can potentially also elicit anti-inflammatory effects in other cell types beyond microglia, we assessed NO production in RAW 264.7 macrophage cells and also in primary astrocyte cultures in the presence and absence of pretreatment with the compound. Securinine was found to dose-dependently decrease NO production in LPS-stimulated RAW264.7 macrophage cells (Figure 8(a)) and also in primary mouse astrocyte cultures (Figure 8(b)). When astrocytes were stimulated with LPS plus IFN-*γ*, greater levels of NO production were achieved, which were similarly inhibited by the securinine (Figure 8(c)). These results indicated that the small molecule securinine also has anti-inflammatory effects in peripheral macrophages and in astrocytes.

## 4. Discussion

In the present study, we examined the anti-inflammatory properties of the small molecule natural product securinine in glial populations and in macrophages and have delineated the potential signal transduction pathways involved in these processes. Our studies demonstrate that securinine strongly inhibits the inflammatory activation of microglia, astrocytes, and macrophages. Securinine significantly and dose-dependently reduced NO production in LPS-stimulated BV2 microglial cells and primary microglia and astrocyte cultures. NF-*κ*B and MAPK pathways were at least partly involved in the anti-inflammatory mechanisms of securinine. Furthermore, securinine also inhibited IFN-*γ*-induced NO production and STAT1*α* activation. Finally, conditioned media from securinine pretreated BV2 microglial cells significantly reduced dopaminergic neurotoxicity compared to LPS-treated group alone. These results suggested that securinine might have therapeutic potential for various glial-mediated neuroinflammatory diseases.

Activation of microglial cells, the resident immune macrophage-like cells in the brain, is beneficial during acute infection or toxic insult by eliminating “sick” neurons that are no longer functional. However, in the presence of ongoing progressive brain damage, they can become chronically activated, resulting in sustained aberrant inflammatory response [25]. Glial cell activation and increased production of proinflammatory products derived from them have been implicated in the pathophysiology of several neurodegenerative diseases, such as AD, PD, and HD [26]. Recently, much attention has been paid to therapeutic strategies aimed at inhibiting neurotoxic microglial activation. Although nonsteroidal anti-inflammatory (NSAIDs) medications demonstrate neuroprotection in various disease models, such medications can have serious side effects following their long-term usage, leading to searches for better alternatives [27]. Recent studies have demonstrated that natural products and their components are good alternative candidates for therapeutic purposes due to their reputation for being safe, inexpensive, and readily available [28].

Activated microglia secretes proinflammatory mediators including cytokines such as IL-6 and TNF-*α*, reactive oxygen species, and reactive nitrative species such as NO. Nitric oxide (NO) is a unique biological messenger which mediates several physiological functions. However, under conditions of excessive production, NO appears to be neurotoxic suggesting that NO may play an important role in pathophysiology of neurodegenerative diseases [29]. In this current study, we have demonstrated that production of NO by LPS-activated microglia (both BV2 and primary microglia) is significantly inhibited in a dose-dependent manner by securinine at both the mRNA and protein levels.

We next examined the effects of securinine on LPS-induced activation of various MAPKs as well as NF-*κ*B-mediated pathways. It is well established that MAPKs and NF-*κ*B transcription factors play an important role in regulation of expression of proinflammatory cytokines and enzymes including iNOS, TNF-*α*, IL-1*β*, IL-6, and COX-2 [30]. Therefore, for a compound to exhibit anti-inflammatory effects this should require an ability to attenuate the activation of these pathways. The signaling mechanisms involved in NF-*κ*B activation have been well established and involve a cascade of cytoplasmic proteins leading ultimately to the translocation of the p65 subunit of NF-*κ*B into the nucleus resulting in transcription of downstream proinflammatory genes [31, 32].

In this study, we show, as expected, phosphorylation of all three MAPKs following LPS treatment of BV2 microglia as well as phosphorylation of NF-*κ*B p65 and increased NF-*κ*B promotor activity. Securinine dose-dependently inhibited LPS-induced NF-*κ*B phosphorylation and activation as well as LPS-induced p38, JNK, and ERK phosphorylation in these cells.

Earlier studies have demonstrated that the expression of iNOS is regulated coordinately by the action of several transcription factors such as NF-*κ*B, AP-1, and STAT1 [33]. Interestingly, our results demonstrate that, in addition to attenuating NF-*κ*B activation, securinine also inhibited IFN-*γ*-induced NO production, iNOS mRNA expression, and STAT1*α* activation (Figure 6). These results indicate that securinine regulates proinflammatory gene expression in both NF-*κ*B dependent and independent manner.

Recent studies have emphasized the role of peripheral macrophages infiltration in reactive gliosis following traumatic brain injury (TBI) and also in the case of spinal cord injuries [34]. Furthermore, when the blood-brain barrier is compromised during neurological disorders such as multiple sclerosis and Alzheimer's and Parkinson's diseases, peripheral immune cells including monocytes, neutrophils, T cells, and B cells can enter the brain where they execute distinct cell-mediated effects [35]. Our studies with macrophages and astrocytes further demonstrate that securinine not only inhibits microglial activation but also inhibits peripheral macrophages and astrocytes indicating the broad anti-inflammatory action of securinine.

A recent study reported that securinine can reduce the glial inflammatory responses induced by beta-amyloid protein, improving both cognitive deficits and neurodegeneration in beta AP (25–35)-treated rats [36] Although the study suggested that securinine was neuroprotective in this model, the exact mechanisms involved in this process were not explored. In the present study, cultured media from LPS/securinine-treated microglia provided almost complete dopaminergic neuroprotection compared with CM from LPS-stimulated microglia. Taken in total, our results suggest that securinine offers neuroprotective effects via reducing abnormal production of proinflammatory mediators. Although CM experiments utilizing LPS-stimulated BV2 microglia with primary mesencephalic cultures may not completely mimic the in vivo situation, it likely, at least in part, reflects the pathological condition under which activated microglia can influence the survival of neighboring neurons in the living brain. Further studies are, however, required to validate mechanisms underlying the neuroprotective property of securinine in animal models of inflammation-mediated neurodegenerative diseases including PD.

## 5. Conclusion

In summary, our results demonstrate that the neuroprotective properties of securinine may be due to inhibition of glial activation and subsequent generation of proinflammatory factors. Mechanistically, this appears to involve inhibition of the p38 MAP kinase-NF-*κ*B pathway resulting in reduced expression of proinflammatory genes and release of corresponding gene products.
